# Albuminuria, glycemic variability and effect of flash glucose monitoring based decision making on short term glycemic variability in Indian type 2 diabetes patients: Indi-GlyVar study

**DOI:** 10.3389/fendo.2022.1011411

**Published:** 2022-11-18

**Authors:** Deepak Nathiya, Mahaveer Singh, Supriya Suman, Hemant Bareth, Nikita Pal, Arjav Jain, Balvir S. Tomar

**Affiliations:** ^1^ Department of Pharmacy Practice, Institute of Pharmacy, Nims University, Rajasthan, Jaipur, India; ^2^ Department of Clinical Studies, Fourth Hospital of Yulin (Xingyuan), Yulin, Shaanxi, China; ^3^ Department of Clinical Sciences, Shenmu Hospital, Shenmu, Shaanxi, China; ^4^ Department of Endocrinology, National Institute of Medical Sciences, Nims University Rajasthan, Jaipur, India; ^5^ Institute of Pediatric Gastroenterology and Hepatology, Nims University Rajasthan, Jaipur, India

**Keywords:** glycemic variability, type 2 diabetes, FGMS, albuminuria, diabetic kidney disease

## Abstract

**Aim and scope:**

Glycemic variability (GV) denotes the fluctuations in the glucose values around the baseline. High glycemic variability is associated with a higher risk of diabetes-associated complications. In this study, we sought to determine the impact of therapeutic interventions based on flash glucose monitoring on rapid, short-term glycemic variability. We also studied the prevalent albuminuria in diabetic kidney disease and its effect on glycemic variability.

**Methods:**

In a 14-day, single-center, prospective intervention study, we measured the GV indices at baseline (days 1–4) and ten days after ambulatory glucose profile-based intervention using flash glucose monitoring (Abbott Libre Pro, Abbott Diabetes Care, Alameda, California, USA) in patients with type 2 diabetes. An EasyGV calculator was used to estimate the flash glucose monitoring (FGM)-derived measures of GV. The primary outcome was to assess the impact of FGMS-based therapeutic interventions on glycemic variability markers: SD, mean amplitude of glycemic excursion [MAGE], continuous overall net glycemic action [CONGA], absolute means of daily differences [MODD], *M* value, and coefficient of variance [%CV], AUC below 70 mg/dl, low blood glucose index, AUC above 180 mg/dl [AUC >180], high blood glucose index [HBGI], and J index. Time-related matrices (time in range (%), time above range (%), and time below range (%) were also calculated from the ambulatory glucose profile. Renal function parameters (serum creatinine, estimated glomerular filtration rate, urine albumin excretion) were calculated. The GV with regard to albumin excretion rate was compared.

**Results:**

Fifty-eight T2DM patients (63.8%, males) with a mean age of 51.5 ± 11.9 years were studied. When compared with baseline (days 1–4), on day 14, there was a significant improvement in mean sensor glucose (mg/dl) median (IQR) [155 (116–247) *vs* 131 (103–163) (p ≤0.001)], JINDEX [15,878 (7,706–28,298) *vs* 8,812 (5,545–14,130) (p ≤0.001)], HBGI [361 (304–492) *vs* 334 (280–379) (p ≤0.001)], MAGE (mg/dl) [112 (8–146) *vs* 82 (59–109) (p ≤0.001)], M-value [2,477 (1,883–3,848) *vs* 2,156 (1,667–2,656) (p ≤ 0.001)], MAG (mg/dl) [111 (88–132) *vs* 88 (69–102) (p ≤ 0.001)]. Patients with albuminuria at baseline had high mean sensor glucose (mg/dl) median (IQR) [190 (131–200) *vs* 131 (112–156) (p = 0.001)], CONGA (mg/dl) median (IQR) [155 (101–165) *vs* 108 (83–120) (p = 0.001)], JINDEX, HBGI, MAGE (mg/dl), and M-value are, median (IQR) [20,715 (10,970–26,217 *vs* 91,118 (6,504–15,445)) (p ≤ 0.01)], [415 (338–423) *vs* 328 (292–354) (p = 0.001)], [125 (102–196) *vs* 103 (74–143) (p ≤ 0.01)], [3,014 (2,233–3,080) *vs* 2,132 (1,788–2,402) (p ≤0.01)], respectively.

**Conclusion:**

In type 2 diabetes, flash glucose monitoring-guided therapeutic interventions can reduce glycemic variability in a brief span (10 days) of time. Also, albuminuria in type 2 diabetes is associated with high glycemic variability. Reduced diabetes complications may ultimately result from this reduced glycemic variability.

## Introduction

In 2021, the International Diabetes Federation (IDF, 10th edition Atlas) estimated the prevalence of diabetes among adults (20–79 years) at around 9%, with 537 million in total. By 2045, this could reach 784 million, with a disproportionately high burden in the Southeast region and middle- to low-income countries (68% *vs*. 46%) (1). If type 2 diabetes is not controlled, diabetic complications include chronic kidney disease (CKD) (40%), ischemic heart disease (30%), cataracts (20%), retinopathy (15.4%), peripheral vascular disease (11.5%), and cerebrovascular accidents (CVAs) (6.9%), will develop in long-term (2–4).

Glycated hemoglobin (HbA1c), a marker of glycemic control, does not account for the fluctuation in blood glucose values. Glycemic variability (GV) matrices are believed to be a more accurate indicator of glycemic control. The American Diabetes Association also recommended time measures (time in range, time above range, and time below range) to evaluate glycemic control (5). The GV was added to the control parameter because variations in glucose levels raise reactive oxygen species (ROS) and impair endothelial function (6, 7). This endothelial dysfunction increases the prevalence of renal (albuminuria, CKD) and atherosclerotic vascular diseases. For the various parts of the glycemic spectrum, several GV measures are utilized, including SD, MAGE, MAG, JINDEX, LBGI, and HBGI.

The ambulatory glucose profile (AGP) and glycemic variability (GV) are frequently generated using retrospective flash glucose monitoring (FGM). The therapy of type 2 diabetes is frequently determined by several clinical factors (age, comorbidities, life expectancy, and risk of hypoglycemia) without considering baseline glycemic variability. This blinded approach may not correct the glycemic variability. Even in cases of well-controlled diabetes, this untreated GV may be the root of diabetic complications. The glycemic variability is influenced by diet, exercise, and drugs. The data regarding the single use of ambulatory glucose and GV-based therapeutic amendments have not yet been studied.

In this study, we tried to see the effect of retrospective FGMS-based decision-making on short-term glycemic variability.

## Material and methods

### Study design

This single-center prospective intervention study was conducted from March 2021 to August 2021 in the endocrinology outpatient department (OPD) of the National Institute of Medical Sciences and Research Hospital, Rajasthan, Jaipur, India. The protocol was developed under the principles of the Declaration of Helsinki and approved by the Institutional Ethics Committee (IEC No. NIMSUR/IEC/2021/0112). We obtained informed consent from the study participants.

### Study population

The study enrolled patients aged 18 to 70 with type 2 diabetes mellitus and HbA1c levels ranging from 6.5 to 11.5%. We excluded patients with advanced diabetic complications. The following were the exclusion criteria: Serum creatinine >1.5 mg/dl, eGFR 30 ml, severe NPDR (Non-Proliferative Diabetic Retinopathy) or higher, including PDR (Proliferative Diabetic Retinopathy), uncontrolled CAD (coronary artery disease), angina, and heart failure. We also excluded immunocompromised individuals, patients with active malignant disease, and patients with chronic conditions such as heart failure, cognitive disorders, dementia, amnesia, autoimmune diseases, drug addiction, pregnancy, and breastfeeding females.

### Collection of the demographic details and clinical data

We obtained demographic information about the patients, such as their age, gender, place of residence, and social status. We also obtained clinical data on diabetes duration, anti-diabetic medicines, comorbidities, and complications.

### Anthropometric measurements

We measured height and weight and calculated the body mass index (BMI). The waist circumference and hip circumference were measured using flexible fiberglass tape. The waist–hip ratio (WHR) was determined using the above two measures.

### Blood investigations

Glycosylated hemoglobin (HPLC, Bio-Rad 2, Alfred Noble Drive, Hercules, California, USA) was used to assess glycemic control. We measured blood urea, serum creatinine, urine albumin creatinine ratio (UACR), serum bilirubin, serum albumin, serum AST, and ALT (HUMAN analyzer, Gesellschaft für Biochemical and Diagnostic GmbH Wiesbaden, Germany) to diagnose diabetes complications and end-organ dysfunction. We performed a direct fundus examination to rule out diabetic retinopathy. We performed a fasting lipid profile (HUMAN analyzer, Gesellschaft für Biochemical and Diagnostic GmbH Wiesbaden, Germany) to assess diabetes-associated dyslipidemia.

The patients were categorized as the urine albumin creatinine ratio. If the fasting morning spot is done, then albumin excretion <30 mg/g of creatinine is considered normal.

### Flash glucose monitoring system

The Abbott FreeStyle Libre Pro Flash Glucose Monitoring System (FGMS) (Abbott Diabetic Care, Alameda, California, USA) was used to calculate glycemic variability. Abbott FreeStyle Libre Pro is a retrospective tool that can be used for 14 days. The Abbott Libre Pro’s MARD (mean absolute relative difference) is 10.1% (80–180 mg/dl—10.7%, >180 mg/dl—8.7%, 70 mg/dl—14%). Compared to the YSI reference, 99.9% of the glucose values were in the Consensus Error Grid Zones A and B.

### Sensor insertion and GV calculation

After obtaining the patient’s consent, we inserted the sensor. Patients were advised to continue with the same diet, exercise, and diabetes medications for four days before returning to the OPD. Patients were asked to come on the fifth day after four days to collect baseline GV data. The variables of the glycemic variability were also derived by the excel-based calculator (EasyGV, Nuffield Primary Care Department, University of Oxford, Radcliffe Primary Care Building, Radcliffe Observatory Quarter, Woodstock Road, Oxford, OX2 6GG, United Kingdom). These variables were MAGE (mean average glycemic excursion), SD (standard deviation), HBGI (high blood glucose index), LBGI (low blood glucose index), M-value, CONGA (continuous overall net glycemic action), MAG (mean absolute glucose), MODD (mean of daily differences), J-index, and LI (lability index). We discussed ambulatory glucose profile patterns with the patients. The AGP guided the therapeutic changes (diet, exercise, and medicines). The patients were counseled about nutrition using a food frequency questionnaire. They were given a standard diabetic diet chart using the plate technique adapted from the ICMR Nutritive Value of Indian Foods, National Institute of Nutrition, Hyderabad.

### FGMS-based therapeutic amendments and follow up

On day five, the following interventions were based on the ambulatory glucose profile and guided by the diet and exercise log.

- Meal and post-meal excursions were analyzed. The meal pattern was altered if the glycemic variability was caused by an irregular diet, such as poor meal timing or content.
- The anti-diabetic medication was modified based on the ambulatory glucose profile and trends.
- If physical activity is associated with increased glycemic variability, the kind, frequency, and intensity of the exercise were adjusted as needed. Mid-activity snacks were introduced to prevent hypoglycemia during exercising.

We asked the patients to return after the flash glucose monitoring system was completed (after 14 days). At the time, we downloaded the sensor data and used the EasyGV calculator to calculate the GV matrices (version 9.0.R2 2).

### Statistical analysis

The software IBM Statistical Package for Social Sciences (SPSS) v22 was used for data entry and statistical analysis (IBM Corp. Version 22, Chicago, Illinois, USA).

The mean ± standard deviation (SD) or median (IQR) was used to represent continuous variables, depending on whether the data distribution was parametric or non-parametric, as determined by the Shapiro–Wilk test. The paired t-test was applied to compare pre-post means in parametric data. In non-parametric data, the Wilcoxon signed rank test was applied to compare pre-post means. Pearson’s and Spearman’s correlations were used to determine the association between GV parameters and their determinants. Multiple logistic regression was used to determine the association between GV and UACR. The level of statistical significance was set at p <0.05.

## Results

**Figure 1:**
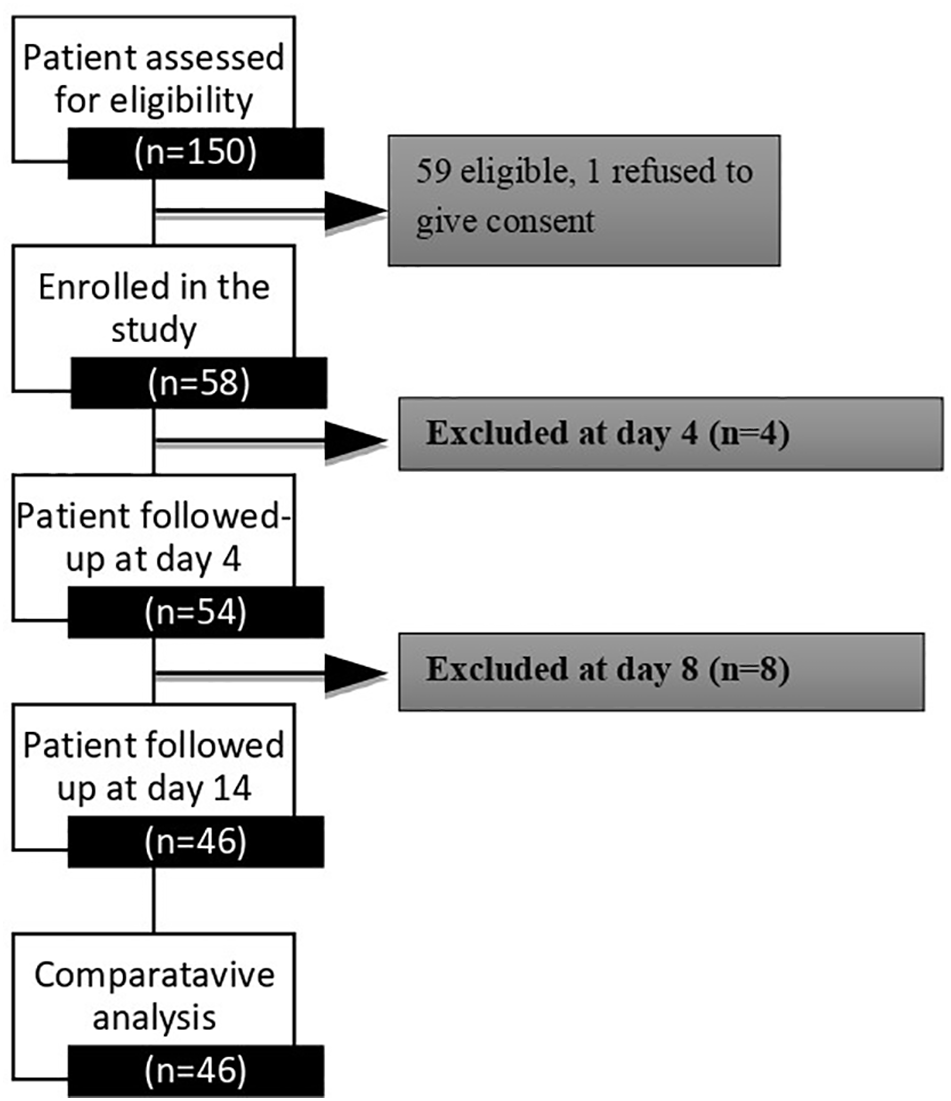
A schematic study plan in consideration of the patients enrolled in the study.

One hundred fifty patients were screened at the Nims Hospital and Research Centre’s Endocrine Outpatient Department, and fifty-nine were found to be eligible. The study flow sheet is depicted in **Figure 1**. Fifty-eight patients were enrolled in the study. Thirteen patients were excluded from the study for a variety of reasons; including patient withdrawal related to logistical issues (n−3), therapeutic modifications (n−3), sensor dysfunction (n−6), and patient refusal to consent (n−1). We have a complete data set of 46 (79.3%) patients at the end of this study.

### Baseline demographics

Fifty-eight T2DM patients (63.8% were males) with a mean age of 51.5 ± 11.9 years were studied. Twenty-five (43.2%) were unemployed or retired, 25 (43.2%) were active smokers, and nine (17.3%) were alcoholics. The detailed demographic variables are shown in **Table 1**.

**Table 1:** Baseline demographic variables of the patients, showing profession and social status.

Variables	Values
Age [mean ± Std. Error] (yrs)	51.47 ± 11.89
**Gender [% (n)]**	
Male	63.8 (37)
Female	36.2 (21)
**Occupation [% (n)]**	
Professional	10.3 (6)
Semi-professional	8.6 (5)
Clerical, Shop owner farmer	17.3 (10)
Skilled worker	10.3 (6)
Semi-Skilled worker	6.9 (4)
Unskilled Worker	3.4 (2)
Unemployed	43.2 (25)
**Social Economic Status [% (n)]**	
Lower Class	5.2 (3)
Upper Lower Class Lower Middle Class	22.4 (13) 39.7 (23)
Upper Middle Class	22.4 (13)
Upper Class	10.3 (6)
**Education [% (n)]**	
Graduate/Postgraduate	7.2 (10)
Intermediate/Post-high School diploma	10.3 (6)
High School	10.3 (6)
Middle School	12.2(7)
Primary School	17.2 (10)
Illiterate	32.8 (19)

### Glycemic variability and albumin excretion

We compared the glycemic variability in patients with normal and abnormal UACR and found that mean sensor glucose (mg/dl) [median (IQR)] [130 (110–150) *vs* 189 (138–195) (p = 0.001)], CONGA (mg/dl) [median (IQR)] [104 (81–118) *vs* 152 (109–164) (p ≤0.01)], JINDEX [median (IQR)] [9,118 (6,504–15,445) *vs* 20,715 (10,970–26,217) (p ≤0.01)], HBGI [median (IQR)] [328 (292–354) *vs* 415 (338–423) (p = 0.001)], MAGE (mg/dl) [median (IQR)] [103 (74–143) *vs* 125 (102–196) (p ≤0.01)], and M-value [median (IQR)] [2,132 (1,788–2,402) *vs* 3,014 (2,233–3,080) (p ≤0.01)] were significantly higher in the patients with abnormal UACR. **Table 2** illustrates this.

**Table 2:** A comparison of glycemic variability in patients with normal and abnormal urine albumin excretion ratios revealed that patients with high albumin creatinine ratios had poor glycemic variability.

Determinants	Urine albumin excretion rate (mg/day)			Urine albumin creatinine ratio (mg/g)		
	Normal [median (IQR)]	Abnormal [median (IQR)]	*p-Value*	Normal [median (IQR)]	Abnormal [median (IQR)]	*p-Value*
**AGE (Years)**	51 ± 12	**51** ± 9	0.53	53.1 ± 12	**49** ± 10	0.14
**MALE [% (n)]**	36 (n = 21)	22 (n = 13)	0.83	32 (n = 19)	**25 (n = 15)**	0.84
**BMI (kg/m^2^)**	25.8 ± 4.7	26.9 ± 4.7	0.33	26.4 ± 4.3	26 ± 5.3	0.96
**WHR**	0.92 ± 0.09	0.97 ± 0.07	0.20	0.93 ± 0.08	0.95 ± 0.08	0.56
**MEAN (mg/dl)**	**131 (112–156)**	**190 (131–200)**	0.01*	**130 (110–150)**	**189 (138–195)**	0.001**
**SD (mg/dl)**	43 (36–65)	57 (39–76)	0.08	**41 (35–64)**	**63 (43–89)**	<0.01**
**CV (%)**	34 (27–40)	33 (32–38)	0.87	32 (26–39)	36 (32–45)	0.87
**CONGA (mg/dl)**	**108 (83–120)**	**155 (101–165)**	0.02*	**104 (81–118)**	**152 (109–164)**	<0.01**
**LI**	2,711 (1,842–4,690)	3,185 (2,140–7,105)	0.23	**2,705 (1,619–4,490)**	**3,596 (2,543–7,399)**	0.01*
**JINDEX**	**16,447 (9,264–26,898)**	**19,499 (9,468–26,431)**	0.03*	**9,118 (6,504–15,445)**	**20,715 (10,970–26,217)**	<0.01**
**HBGI (mg/dl)**	**332 (295–354)**	**415 (329–429)**	<0.01**	**328 (292–354)**	**415 (338–423)**	0.001**
**MAGE (mg/dl)**	110 (81–150)	115 (89–174)	0.14	**103 (74–143)**	**125 (102–196)**	<0.01**
**M-VALUE**	**2,162 (1,813–2,469)**	**3,029 (2,133–3,154)**	<0.01**	**2,132 (1,788–2,402)**	**3,014 (2,233–3,080)**	<0.01**
**MAG**	88 (79–104)	89 (77–112)	0.29	**87 (78–101)**	**94 (80–117)**	0.01*
**TIR (%)**	74 (46–87)	52 (31–77)	0.05	**76 (58–88)**	**52 (30–76)**	<0.01**
**TAR (%)**	**35 (12–68)**	**48 (16–66)**	0.01*	**11 (4.5–33.7)**	**48 (18–66)**	<0.01**
**TBR (%)**	**15 (9–27)**	**0.5 (0–6)**	0.01*	8.5 (1–13.5)	1 (0–6)	0.16
**HbA1C (%)**	7.6 (6.2–9.5)	8.0 (6.7–11.7)	0.05	**7.5 (6.1–9.8)**	**8.6 (6.8–11.4)**	<0.01**
**NET AUC>180 (mg/dl/min)**	90,735 (30,975–375,075)	545,407 (74,032–874,241)	0.06	**73,245 (27,427–346,106)**	**607,245 (97,920–827,685)**	<0.01**
**NET AUC<70 (mg/dl/min)**	**16,860 (1,387–33,382)**	**1,432 (78–7,353)**	0.01*	15,232 (435–68,296)	2,010 (690–12,135)	0.26

The bold values represent the significant values, we have used them to highlight values. *p-value<0.05; **p-value <0.01.

The UACR correlated with the mean sensor glucose (mg/dl) (0.527) (p = 0.001), CONGA (mg/dl) (0.501) (p = 0.01), JINDEX (0.529) (p = 0.001), HBGI (0.521) (p = 0.001), MAGE (mg/dl) (0.502) (p = 0.01), M-value (0.528) (p = 0.001). UACR and GV analysis are shown in **Table 3**.

**Table 3:** Correlation of the spot urine albumin creatinine ratio (mg/g) with glycemic variability variables at baseline (days 1–4).

	MEAN (mg/dl)	SD(mg/dl)	CV (%)	CONGA (mg/dl)	LI	JINDEX	HBGI	MAGE (mg/dl)	MVALUE	MAG(mg/dl)	TIR (%)	TAR (%)	TBR (%)	HbA1C (%)
**UUrine albumin creatinine ratio (mg/g)**	<0.001*** (0.527)	<0.01** (0.511)	00.08 (0.270)	<0.01** (0.501)	<0.01** (0.447)	<0.001*** (0.529)	<0.001*** (0.521)	<0.01** (0.502)	<0.001*** (0.528)	<0.01** (0.442)	<0.01**(-0.496)	<0.001*** (0.518)	00.33 (-0.150)	00.01* (0.352)

The bold values represent the significant values, we have used them to highlight values. *p-value <0.05; **p-value <0.01; ***p-value <0.001.

### Effect of FGMS-based therapeutic amendments on short-term glycemic variability

**Figure 2:**
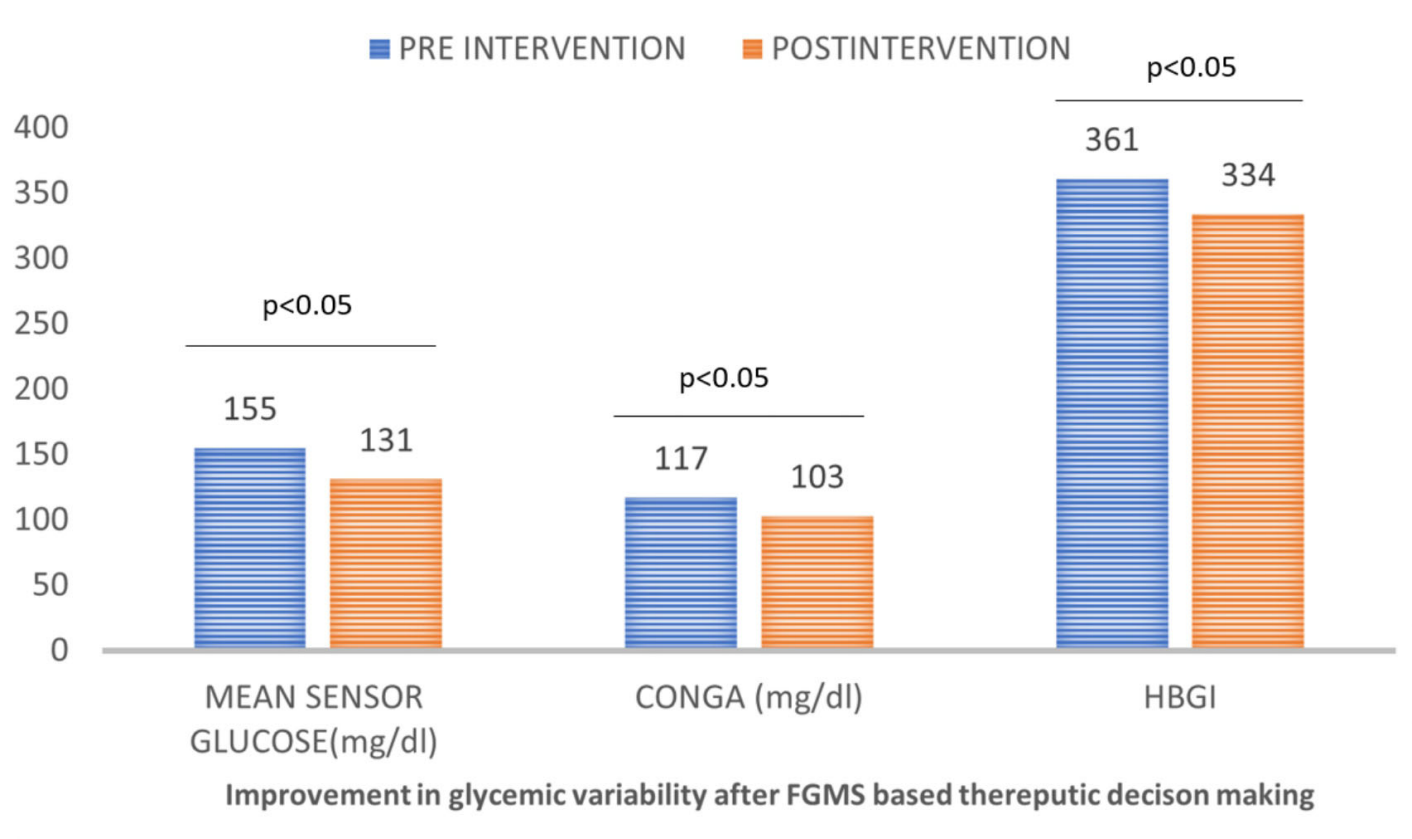
Effect of FGMS based therapeutic decision making on short-term glycemic variability.

We assessed glycemic variability at baseline (days 1–4), generated (diet, exercise, and drugs) on day 5, and subsequently measured GV on day 14. The glycemic variability between day fourteen (after the intervention) and baseline (days 1–4 before the intervention) was compared. There was a significant improvement in mean sensor glucose (mg/dl) median (IQR) [155 (116–247) *vs* 131 (103–163) (p ≤0.001)], JINDEX [15,878 (7,706–28,298) *vs* 8,812 (5,545–14,130) (p ≤0.001)], HBGI [361 (304–492) *vs* 334 (280–379) (p ≤0.001)], MAGE (mg/dl) [112 (8–146) *vs* 82 (59–109) (p ≤0.001)], M-value [2,477 (1,883–3,848) *vs* 2,156 (1,667–2,656) (p ≤0.001)], MAG (mg/dl) [111 (88–132) *vs* 88 (69–102) (p ≤0.001)]. CV, on the other hand, was numerically lower but failed to achieve statistical significance. This is shown in **Table 4** and **Figure 2**.

**Table 4:** The effect of FGMS-based therapeutic decision making on short-term glycemic variability.

Glycemic Variability Indices	Baseline (Days 1–4)(Pre-intervention^#^)Median (IQR)	Day 14 (Post-intervention)Median (IQR)	p-Value
Mean sensor glucose (mg/dl)	155 (116–247)	131 (103–163)	<0.001***
Standard deviation of glucose (mg/dl)	46 (32–62)	32 (24–51)	<0.01**
Coefficient of Variation (%)	28 (23–32)	27 (21–34)	0.109
Continuous overall net glycemic action (CONGA) (mg/dl)	117 (82–201)	103 (76–132)	<0.01**
Lability Index (LI)	4,061 (2,067–6,631)	1,869 (1,171–4,520)	<0.01**
JINDEX	15,878 (7,706–28,298)	8,812 (5,545–14,130)	<0.001***
High blood glucose index	361 (304–492)	334 (280–379)	<0.001***
Mean amplitude of glycemic excursion (mg/dl)	112 (84–146)	82 (59–109)	<0.001***
M-value	2,477 (1,883–3,848)	2,156 (1,667–2,656)	<0.001***
Mean absolute glucose (mg/dl)	111 (88–132)	88 (69–102)	<0.001***

**p-value <0.01, ***p-value <0.001.

^#^The ambulatory glucose profile and glycemic trends were discussed with the patients on day five.

### Safety profile and product compliance

During the study, no adverse drug reactions were reported. The given products were well received by all patients.

## Discussion

The effect of retrospective flash glucose monitoring-based decision-making on glycemic variability was investigated in this study. The therapeutic changes based on flash glucose monitoring reduced glycemic variability in a very short period. Patients with high glycemic variability also had a high albumin excretion rate.

Poor GV carries a high risk of diabetic complications, specifically cardiovascular disease. Twenty to forty percent of type 2 diabetes patients have albuminuria (8–10). Patients with obesity, hypertension, and dyslipidemia are more likely to have albuminuria. Additionally, albuminuria has been linked to a higher risk of cardiovascular disease (11). This synergy exists because glycemic variability is associated with endothelial dysfunction, and albuminuria reflects endothelial dysfunction. In one study, patients with a stroke and a high J-index at the time of admission had an increased risk of cardiovascular death and 3-P MACE (12). GV is associated with myocardial damage and predicts mortality in patients with myocardial infarction (13). This proves that endothelial dysfunction plays a central role in cardiovascular and renal diseases.

The flash glucose monitoring system improves doctors’ therapeutic decision-making (drugs, diet, and exercise) over traditional blood glucose self-monitoring by producing the ambulatory glucose profile and glycemic patterns. In an Indian multi-centric study, 181 patients with type 2 diabetes were studied using iPro-2 retrospective CGMS. Although the glycemic variability matrix was not studied, they demonstrated that the therapeutic change based on the overlay resulted in diabetes improvement (14). On the other hand, we used retrospective FGMS and adjusted therapy accordingly, resulting in rapid control of GV.

This is the first study of its kind to investigate glycemic variability and the effect of ambulatory glucose profile-based decision-making (diet, exercise, and medicines) on short-term GV. MAGE, which indicates postprandial excursion, is associated with long-term cardiovascular disease. In our study, MAGE improved within ten days. This could lead to improved cardiovascular outcomes.

The study did have some limitations, most of which were due to COVID-19. The sample size was small, and the study was brief. There is also a lack of long-term data to assess the impact on diabetic complications. However, we expect the complications to decrease over time as glycemic variability decreases. A more comprehensive study is needed to determine the impact on diabetic complications.

## Conclusions

High glycemic variability is linked to albuminuria in type 2 diabetes or vice versa. In a very short period, a treatment intervention based on flash glucose monitoring decreased the glycemic variability (10 days). A longer follow-up is needed to see the effect on diabetic complications.

## Data availability statement

The raw data supporting the conclusions of this article will be made available by the authors, without undue reservation.

## Ethics statement

The studies involving human participants were reviewed and approved by the Institutional Ethical Committee, Nims University Rajasthan. The patients/participants provided their written informed consent to participate in this study.

## Author contributions

MS: Conceptualization, methodology, investigation, validation, and original draft writing. DN: Methodology, project administration, and supervision. SS: Investigation, writing, reviewing, and editing. HB: Investigation, methodology, reviewing, and editing. NP: Investigation, formal analysis, original draft writing. AJ: Investigation, data interpretation, formal analysis, and original draft writing. BT: Conceptualization, supervision, resources, funding, and critical review. All authors contributed to the article and approved the submitted version.

## Funding

The funding for FGMS was provided by the NIMS University Rajasthan, Jaipur, India. There was no other funding received from any other sources.

## Conflict of interest

The authors declare that the research was conducted in the absence of any commercial or financial relationships that could be construed as a potential conflict of interest.

## Publisher’s note

All claims expressed in this article are solely those of the authors and do not necessarily represent those of their affiliated organizations, or those of the publisher, the editors and the reviewers. Any product that may be evaluated in this article, or claim that may be made by its manufacturer, is not guaranteed or endorsed by the publisher.
